# Who Shall Enforce the Dental Law?

**Published:** 1877-05

**Authors:** 


### EDITORIAL.

### WHO SHALL ENFORCE THE DENTAL LAW?

We have frequent enquiries from dentists in Ohio, in reference to carrying out the provisions of the law for regulating the practice of dentistry in the State. These enquiries usually have reference to prosecution for violation of the law. Many seem to think it the business of the Board of Examiners. Upon examination it will be found, that the duties of the Board of Examiners are clearly defined in the law, and that prosecuting violators of the law is not one of their duties. Their duty consists in examining candidates, and granting certificates of qualification to those who satisfactorily pass the examination. With that the duties of the Board, as such, end. They have nothing more to do in the prosecution of offenders of this law than any other citizen, and no more certainly than any other member of the profession. Some have suggested that it was the duty of the Executive Committee of the State Dental Society. No such duty is imposed upon that Committee. It has no more business with this matter, than it has to ferret out and prosecute horse-thieves.

It is the duty of any one who may be cognizant of violations of law, to see that the offenders are properly dealt with, and made to respect the law; and especially is this the duty of every dentist who has conformed to the requirements of the law.

The law is explicit as to the mode of its execution.

SECTION 2. says: "Any person who shall practice Dentistry without having complied with the regulations of this act, shall be deemed guilty of a misdemeanor, and upon conviction thereof, shall be fined not less than fifty dollars nor more than two hundred dollars."

SECTION 3. "All prosecution under this act shall be by indictment before the Court of Common Pleas in the County where the offense was committed."

This means business for any body who wishes to protect Society from the abuses of quackery, and to maintain the usefulness, integrity and honor of the dental profession.

Information of violation of the law, with the names of witnesses, can be given by any one, having such information to the prosecuting attorney of the County, in which the offense has been committed, and it will then receive attention, if the officer is faithful.

There is a hesitancy or delicacy on the part of many in the profession, about taking any active part in enforcing the law, Why is this? Men are bold enough to violate the law. Are there none with sufficient stamina to sustain it? Are the members of the dental profession too feeble to resist quackery, or too timid to use the instruments that have been put into their hands for the protection of the public, and their own defense?

No dentist who has complied with the law, can afford, neither for his own sake nor that of the public, to permit its violation; especially by those whose inclinations and practice are to degradation.

Every dentist, who has the best interests of his profession and society at heart, ought to use his best efforts. to have his professional neighbors possess at least a legal qualification.

